# Bubo in the Pelvis

**Published:** 1844-02-10

**Authors:** 


BUBO IN THE PELVIS.

This is a case very little spoken of in books, and still more rarely met with in practice.

B. Poulonga, aged eighteen, had about six weeks ago a chancre, which presented no particular complication till, after a day of fatigue, he experienced a severe pain in the right iliac fossa. It must be remarked that the chancre occupied the corresponding place at the base of the prepuce, namely, the right side. The pain in the iliac region increased so much, that he was obliged to discontinue his work, and he passed a fortnight without sleep.

When he came into the hospital, the patient had very high fever, with a vague tumefaction, very painful, in the right iliac fossa; the least pressure was insupportable; the stretching out of the limb impossible, and all the movements of the body, especially coughing, were felt most painfully at the seat of the disease.

For some days before his admission the chancre disappeared. Forty leeches to the swelling; emollient drinks; rigid abstinence. The next day he declared himself better; twenty leeches were again applied. After some days the abdominal parietes had recovered their suppleness about the swelling, and hard ganglions could be felt, without pain, above Poupart's ligament, and in the iliac fossa. At the end of ten days the patient was perfectly cured.—*Prov. Med. Jour., from Gaz. des Hopitaux*, Nov. 7., 1843.

